# An Investigation on Modifiable and Nonmodifiable Estrogen Exposure and Gray Matter Volume in Healthy Older Women

**DOI:** 10.1177/26884844251379409

**Published:** 2025-09-15

**Authors:** Heather Kwan, Cassandra Szoeke, Ashleigh Parker, Jodie R. Gawryluk

**Affiliations:** ^1^ University of Victoria, Victoria, British Columbia, Canada.; ^2^ Institute on Aging and Lifelong Health, University of Victoria, Victoria, British Columbia, Canada.; ^3^ University of Melbourne, Melbourne, Victoria, Australia.

**Keywords:** gray matter volume, healthy aging, women’s health, estrogen, hormone therapy

## Abstract

**Introduction:**

It is projected that the global population of adults above age 60 years will surpass 2 billion by 2050. Age-related cognitive decline represents a prevalent issue and research has demonstrated that women are at greater risk than men. Changes in cognitive function with age are influenced by many factors and may include lifetime exposure to estrogen and the transition to menopause. While the exact relationship between estrogen and the aging brain is unclear, the hormonal changes in menopause have been associated with a decline in gray matter volume. However, some studies have demonstrated that the use of hormone therapy may mitigate some of the effects of cognitive decline.

**Methods:**

The current study used magnetic resonance imaging and voxel-based morphometry to examine the relationship between gray matter volume and endogenous lifetime estrogen exposure (*e.g.,* reproductive period length or age of menopause − age of menarche in years) and differences in gray matter volume between women who used hormone therapy (*N* = 62, M_age_ = 70.97 [2.97], M_edu_ = 12.43 [3.22]) and those who did not (*N* = 62, M_age_ = 70.14 [2.62], M_edu_ = 12.81 [3.75]). It was hypothesized that higher lifetime estrogen exposure and use of hormone therapy would be correlated to greater gray matter volume. The data were retrieved from the Women’s Healthy Ageing Project.

**Results:**

Results demonstrated no significant correlations between whole brain gray matter volume and lifetime estrogen exposure. There were no significant differences between groups based on hormone therapy use. However, there was a nonsignificant relationship that suggested that women who did not use hormone therapy had greater gray matter volume than those who did use it.

**Discussion:**

As the aging population continues to grow globally, it is essential to better understand the variables that influence trajectories of aging; especially for women, who are particularly at risk for age-related cognitive decline.

## Introduction

The aging population is growing globally, at an unprecedented rate. The number of women who are perimenopausal or post-menopausal are estimated to reach 1.2 billion by the year 2030, underscoring the need to understand women’s healthy aging.^1^ Fundamentally, understanding the influencing variables among healthy aging individuals, including endogenous and modifiable variables, may lead to increased understanding of brain health trajectories.

Structural changes in the healthy aging brain can be observed using high resolution neuroimaging methods such as magnetic resonance imaging (MRI).^2^ Brain volume decreases with age, and it correlates to poorer cognitive performance.^2–4^ Similar to overall brain volume, white and gray matter both decrease with age, particularly in subcortical structures like the medial temporal lobes and the prefrontal regions.^4^ There is a degree of changes to structure and function of the aging brain that is expected and normative. However, there is also a large amount of individual variability in aging trajectories that largely relates to risk and protective factors. Theoretical models, such as the Revised Scaffolding Theory of Aging and Cognition consider in detail how immutable and modifiable risk factors may contribute to neural resource enrichment or neural depletion.^5^ However, despite sex differences in hormonal changes with age and in risk for age-related neurodegenerative disorders, sex and gender variables have not been incorporated in such models to date.

### Women’s aging

Estrogen is a critical part of human biology and is considered to be neuroprotective; it is both a primarily reproductive hormone and assists in the regulation of dendritic growth.^6^ In women, estrogen levels vary across the lifespan, particularly during the reproductive window; both the fluctuations throughout the menstrual cycle and the impact of larger physiological changes such as puberty, pregnancy, and menopause.^7^ The reproductive window is considered the time between menarche and menopause for biological females. However, it can be impacted by additional factors such as, breastfeeding, pregnancies, hormone therapy, surgical menopause, contraceptives, and antiestrogen therapies.^6,8^ Taken together, the natural variation of estrogen and the internal factors that influence estrogen levels can be referred to as a lifetime exposure of estrogen. In the context of the current study, we examined the number of years that a biological female is exposed to endogenous estrogen, or the reproductive window (age of menopause-age of menarche in years). Internal and external factors can broadly be broken up into endogenous, natural production of estrogen, and exogenous, artificial introduction of estrogen. While both of these factors can be modified to some degree, exogenous factors (like menopausal hormone therapy) tend to be more directly modifiable than endogenous factors. It has been suggested that the lifetime exposure of estrogen can uniquely mitigate the negative consequences associated with cognitive aging. A longitudinal study examined women from the Cache County study and examined their lifetime exposure of estrogen to their cognition.^6^ They found that women with longer reproductive windows had better cognitive health. Another study of women from the UKBioBank found that women who had greater endogenous hormone exposure over the lifespan demonstrated less cerebral small vessel disease.^9^ They examined various impacting factors such as pregnancy, oral contraceptive, and hormone therapy use and found that exogenous contributions did not impact their results. A review described the mixed literature and understanding of how estrogen exposure and the contributing factors influence cognition and brain health.^8^ It is clear that factors relating to female biology increase the risk profile of developing neuropathology and there is research to suggest that lifetime exposure of estrogen plays a critical role; however, the directionality is unclear. Despite the growing recognition of the importance of female biology, there is little research on how lifetime estrogen exposure relates to gray matter volume in postmenopausal women.^6–10^

Conde and colleagues described the neuroprotective effect of estrogen and the depletion of estrogen around menopause has a detrimental impact on women’s cognition.^11^ Furthermore, a study investigated the impact of estrogen and hormone therapy in postmenopausal women.^1^ They found that among nearly 6000 participants, any historical use of estrogen therapy was associated with larger gray and white matter volumes. In particular, these findings were significant irrespective of timing of hormone therapy and other risk factors such as cardiovascular risk, genetics, or lifestyle factors. This suggests that estrogen is not only protective of cognition, but neurophysiology as well. Another study examined the neurophysiological impact of menopause.^10^ They found that menopause impacted connectivity, metabolism in higher order cognitive process, and a plateau of gray matter, which was suggested to be a compensatory mechanism. Taken together, the current literature largely indicates a negative impact of menopause on cognition and brain physiology. However, there is some inconsistency in the findings of this area. A review of studies on the hormonal influence on cognitive function described the lack of significant findings between sex hormones and cognition.^12^ These inconsistencies in findings could potentially relate to interindividual differences that impact the efficacy of hormone therapy. Another review described several moderators of hormone therapy efficacy that included age, timing and duration of exposure of hormone therapy, lifestyle factors such as cardiovascular health.^13^ These differences may play a significant role in the efficacy of hormone therapy for cognition. Therefore further research is needed, particularly considering the potential for the increased risk of breast and cervical cancer associated with long term hormone therapy.^1^ Furthermore, it has been suggested that the individual’s perception of menopause may impact their subjective experience of cognition. A study of women in peri-menopause found that had subjective memory complaints demonstrated a positive correlation with their negative associations with menopause.^14^ Furthermore, these subjective complaints were not indicative of objective deficits on cognitive tests. While there is incongruence in the current literature on estrogen, further investigation is required to understand the impact of menopause.

One possible suggestion for mitigating this particular risk factor is hormone therapy. Although the impact of estrogen has been explored in the literature, the efficacy of hormone therapy on cognition is currently understudied with many citing the importance of continued research in this area.^15^ However, much of the current research on hormone therapy impact is conflicting. For example, one review described that some studies demonstrate cognitive benefits from hormone therapy, other studies show small impacts or none at all.^11^ While another review described the inconsistent impact of estrogen across the lifespan but suggests that the timing of hormone therapy may influences the changes in brain volume that are observed- leading to some of these differences.^7^ For example, some studies have demonstrated that hormone therapy is most useful when used for short periods of time and when it is initiated close to the onset of menopause.^7^ A recent systematic review and meta-analysis examined 34 studies that examined hormone therapy and cognition, and they found that the impact of hormone therapy on cognitive domains differed based on timing and duration of onset.^16^ However, the results are still mixed in the literature and overall conclusions are not definitive.^7^ Overall, the efficacy of hormone therapy on cognition and brain physiology are still undecided and would benefit from further investigation.

The current study aimed to investigate the impacts of endogenous lifetime estrogen exposure as well as the modifiable use of hormone therapy on gray matter volume in aging women. While the literature is currently divided, there is evidence that estrogen is neuroprotective and associated with better neurological outcomes.^6,8,9,17,18^ Therefore, it was hypothesized that increased endogenous lifetime exposure of estrogen, specifically the length of the reproductive period, and use of hormone therapy would demonstrate neuroprotective benefits and be associated with greater volume of gray matter. The ultimate goal of this research is to better understand how sex-specific variables, like endogenous and modifiable estrogen exposure, may influence brain health in aging women.

## Methods

### Participants

The participants of this study were involved in the Women’s Healthy Ageing Project (WHAP); please see https://medicine.unimelb.edu.au/research-groups/medicine-and-radiology-research/royal-melbourne-hospital/healthy-ageing-program#details for additional information. The full set of inclusion and exclusion criteria are described elsewhere.^15^ Inclusion criteria for the current study required participants to have had an anatomical Magnetic Resonance Imaing (MRI) scan during the 2012–2013 follow-up visit and had information for lifetime estrogen exposure and hormone therapy. While the term menopausal hormone therapy is growing within the literature for natural or spontaneous menopausal treatment, the WHAP specifically described this group as using Hormone Replacement Therapy (HRT), therefore HRT users and nonusers will be used to describe participants using menopausal hormone therapy. The study protocol was approved by the Human Research Ethics Board at University of Victoria.

### Measures

#### Lifetime estrogen exposure

Using a similar methodology to previous studies,^6^ the current study focused on HRT nonusers (*n =* 62) and examined the length of their reproductive window to estimate endogenous lifetime estrogen exposure by subtracting the age of menarche from the age of menopause in years. The reproductive window was calculated by subtracting age of final menstrual period from the age of menarche for each participant included in this analysis (M = 41.21 SD = 3.28; [33.62–51.31]). For the instances of missing data from the age of final menstrual period, data were visually inspected, then the instances were calculated to be fewer than 5% of the available data, therefore the data were considered to be missing at random and were corrected using multiple imputation with MICE in RStudio.

#### Hormone therapy

Participants that had used hormone therapy at any time point throughout the study since enrollment were identified (*n =* 62). Participants that had not engaged in hormone therapy were also identified and were manually age- and education-matched to the HRT group (*n* = 62). Individuals who had experienced a hysterectomy or ovariectomy were excluded from the analysis. The purpose for these exclusions was to compare the impact on gray matter from the use of modifiable hormone therapy in “typical” or un-aided menopausal transition, as opposed to surgical menopausal transition and to focus on healthy aging women who received HRT in relation to their menopausal symptoms and not due to surgical post-treatment care.

### Imaging

The images were acquired on a 3.0T Siemens Trim Trio scanner as part of the 2012 WHAP cohort follow up period. Anatomical images were obtained sagittally with a T1-weighted magnetization prepared rapid gradient echo (MPRAGE) MRI with the following parameters: repetition time (TR) of 2300 ms, and echo time (TE) of 2.98 ms, voxel size of 1 mm^3^, and a flip angle of 9 degrees. Diffusion-weighted images were acquired using an echo planar imaging (EPI) sequence with the following parameters: TR of 8500 ms, TE of 90 ms, voxel size of 2.5 mm^3^, Field of View of 240 mm, b value = 0 or 1000 s/mm^2^, total slices = 55, and 30 diffusion directions. Images were stored in a DICOM format and were converted to NIFTI format using dcm2niix in the MRIcroGL application.^19^

### Statistics

All analysis steps were executed with the tools within the fMRI of the Brain Software Library (FSL) version 6.0.5.2. Voxel-based morphometry (VBM) was used to examine the relationship between gray matter volume on a voxel by voxel basis (where voxels represent 2 mm^3^ regions of brain tissue) and measurements of interest. Specifically, structural data were analyzed with FSL-VBM,^20^ http://fsl.fmrib.ox.ac.uk/fsl/fslwiki/FSLVBM), an optimized VBM protocol^21^ carried out with FSL tools.^22^ First, the images were stripped of nonbrain tissue (*i.e.,* skull and meninges) using an automated brain extraction tool. The images were then manually inspected and adjusted for individual accuracy. Next, each individual image was segmented into gray matter, white matter, and cerebral spinal fluid and the individual gray matter images were registered to standardized MNI 152 space.^23^ The resulting images were averaged and flipped along the x-axis to create a left-right symmetric, study-specific gray matter template. This step creates a template or image of only gray matter. Next, all native gray matter images were nonlinearly registered to this study-specific template and “modulated” to correct for local expansion (or contraction) due to the nonlinear component of the spatial transformation. This step allows for each participants’ scan to be “fitted” into the study template for analysis. The modulated gray matter images were then smoothed with an isotropic Gaussian kernel with a sigma of 3 mm. Finally, voxel-wise GLM was applied using permutation-based nonparametric testing. This step allows for GLM analysis across individual voxels while accounting for multiple comparisons. The first analysis examined the correlation between gray matter volume and lifetime estrogen exposure (controlling for age). A second analysis examined differences in gray matter volume between the HRT and non-HRT groups using *t*-tests. The analyses examine the relationship across each voxel in the brain; therefore, all analyses were corrected for multiple comparisons across space with threshold free cluster enhancement.^24^ The resulting images were then examined at a *p* < 0.05 threshold.

## Results

Participants included 124 healthy individuals from the WHAP. All participants were biologically female as identified *via* self-report upon enrollment in the study, participant demographics are included in Table 1. The HRT users and HRT non-users were matched on age [*t*(122) = −1.64, *p* = 0.52] and education [*t*(122) = −0.60, *p* = 0.27].

**Table 1.** Participant Demographic Information

	*N*	Mean	Standard deviation	Range
HRT users age	62	70.97	2.97	66–76
HRT users education	62	12.43	3.22	7–20
HRT nonusers age	62	70.14	2.62	66–76
HRT nonusers education	62	12.81	3.75	6–22

Participant demographics for each analysis. Including number of participants included in each analysis, their mean, standard deviation, and range in years.

### Lifetime estrogen exposure

An examination of the correlation between lifetime estrogen exposure, the reproductive period length, and gray matter volume did not yield a significant result at a threshold of *p* < 0.05 (corrected).

### Hormone therapy

**FIG. 1.**
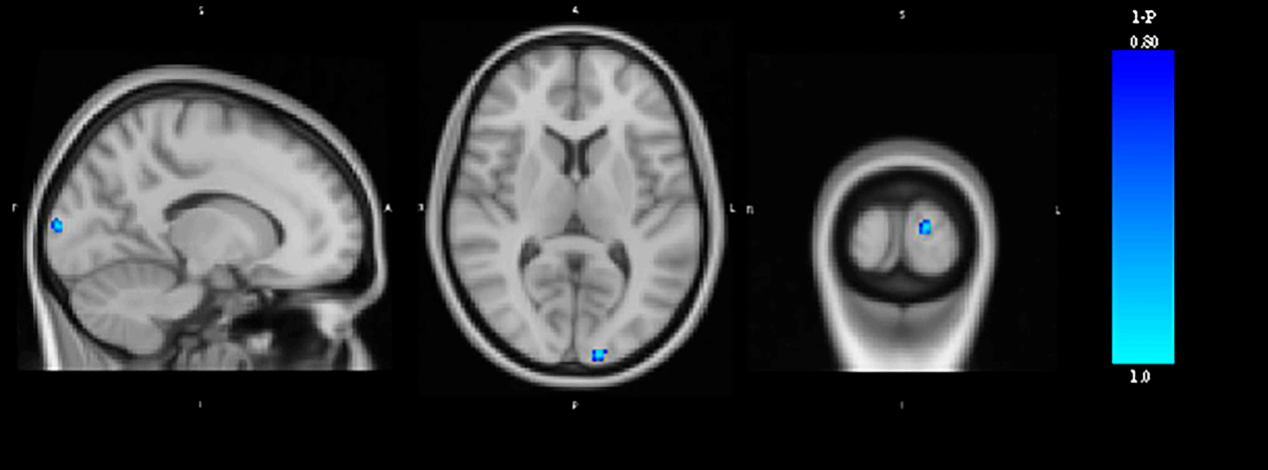
The regions where the HRT nonusers group had greater gray matter volume than the HRT group at a threshold of *p* < 0.2. Images demonstrate in the coronal, sagittal, and transverse plans with an intensity bar to depict the strength of the correlation at each voxel. Images are depicted on an MNI atlas.

The HRT group was compared with the HRT non-users group on gray matter volume using *t*-tests and corrected for multiple comparisons. Neither group demonstrated significantly greater gray matter volume than the other at a threshold of *p* < 0.05 (corrected). However, the HRT nonusers group also showed a small cluster of greater gray matter in the occipital pole compared with the HRT group that did not reach significance (*p* < 0.2, corrected). Table 2 outlines the regions and their significance values. Figure 1 depicts the subthreshold regions where HRT nonusers showed greater gray matter volume than HRT users across all participants.

**Table 2.** HRT Users vs HRT Nonusers Gray Matter Volume Comparison

Cluster size (voxels)	Brain region	Laterality	MNI coordinates			*p* value
			X	Y	Z	
26	Occipital pole	L	−12	−100	8	0.157

The regions where the HRT nonusers group had greater gray matter volume than the HRT group at a threshold of *p* < 0.45. The table demonstrates brain region, laterality, voxel coordinates of the strongest voxel in the cluster in standard space, and the Z-score.

## Discussion

As the aging female population continues to grow, it is critical to examine the modifiable and nonmodifiable factors that may influence their neurological trajectories. The current study aimed to examine the differences in gray matter dependent on sex-specific factors including lifetime estrogen exposure and hormone therapy in healthy older women.

Our first objective was to examine the relationship between endogenous lifetime estrogen exposure, the time between menarche and menopause, and gray matter volume in healthy older women. We hypothesized that increased lifetime estrogen exposure would be positively correlated to gray matter volume. Our results did not demonstrate a significant relationship between gray matter volume and endogenous lifetime estrogen exposure. These results were unexpected given the some of the previous literature. For example, a review described studies where a longer reproductive span was positively associated with cognition and a lower risk of dementia,^8^ but the latter is still debated in the literature. Other studies demonstrate positive influences of hormone therapy, contraception, and reproductive span, in relation to gray matter volume.^25^ However, the overall literature continues to be mixed and there are studies that have also not detected significant relationships within gray matter. For example, one review described that the majority of findings between age of menarche and cognition are null.^8^ However, another study found that estrogen did not have any effect on gray matter volume, but saw significant protective impact in white matter.^17^ Furthermore, other studies have demonstrated significant impacts on other brain level correlates such as white matter.^9^ Generally, the current literature demonstrates mixed findings and the exact relationship between estrogen and the aging female brain remains elusive. With this particular set of participants, there are several possible factors that might have contributed to null findings. First, it is known that there are additional factors that influence lifetime endogenous estrogen exposure that we were not able to include in our analysis (due to data availability) such as breastfeeding and number of pregnancies. By not accounting for these factors, we may have underestimated, or overestimated, the amount of estrogen exposure that women experienced, which may have impacted the results. Second, other studies have examined the relationship between estrogen exposure and white matter or cognition, whereas our results examined gray matter. While gray matter is known to decline with age, it is possible that the estrogen has a greater influence on other neural correlates such as white matter hyper intensities due to the relationship between estrogen and cerebrovascular health,^9^ which could also affect cognition. Functional imaging may also further elucidate the impact of estrogen on the aging brain. Likewise, estrogen was measured using a proxy measure of the lifetime reproductive window but could also be measured using more direct measures such as estradiol. Further research is required to ascertain the exact impact of estrogen exposure in the female aging brain. The current participants were cognitively healthy older women, with no neurodegenerative diagnoses. Future research should examine younger adults and adults impacted by neurodegeneration in order to further examine the influence of estrogen at different stages of the lifespan and neurological development.

Our second objective was to examine the relationship between modifiable estrogen, in the form of hormone therapy, and gray matter volume. We hypothesized that use of hormone therapy would be positively correlated to gray matter volume. There were no significant differences in gray matter volume when comparing the HRT users to the HRT nonusers group in the current study. However, there was a trend toward greater gray matter volume the in posterior regions of the brain (*p* < 0.20, corrected) in women who did not use HRT, compared with those who did. The current findings support some of the current literature; specifically, that hormone therapy does not increase gray matter volume. These findings were contrary to our hypothesis and at odds with some of the current literature as well. In the extant literature, there is often variability in the results of the impact of hormone therapy on gray matter volume.^7^ For example, one study in a study compared a group of 20 women who were taking hormone therapy and 21 women who were not, both with an average age of 57 years.^26^ This study found that the group taking hormone therapy had significantly greater gray matter volume in the parietal and medial temporal areas than the group that did not.^26^ However, another randomized control trial comparing the gray matter volume of 420 participants who were taking hormone therapy and 435 that were taking a placebo found that participants who were taking hormone therapy had significantly less gray matter volume than those who were taking the placebo in the frontal and medial temporal regions of the brain.^27^ A significant limitation to the current study is that the participants were compared based on their historic use of HRT, but not the duration type of use that may have a more pronounced impact on gray matter volume. Future studies should examine the duration of hormone therapy on neurological correlates to better understand this relationship. However, there are several factors that may have influenced our results. The current participants had a large degree of heterogeneity in the duration, type, and reason of use for hormone therapy. A summation of these differences and a list of reasons the HRT nonusers group from the current study did not participate in hormone therapy are listed in Table 3. These differences have the potential to influence the efficacy of hormone therapy and their impact on gray matter volume. Within cognitive data, studies have demonstrated the variable impact of hormone therapy across cognitive domains that is dependent on timing and onset of treatment.^16^ A further complication of this literature on the whole, is that the type, duration, and dosage of hormone therapy is not often described or controlled for—leading to variability across and within studies. The use of hormone therapy does not exist irrespective of other factors and the question of its influence on brain health is increasingly complex. A review several potential moderating factors of hormone therapy impact described that duration of treatment, time of initiation, age, education, and age of menopause all influence hormone therapy efficacy.^7^ Overall, additional research on these nuanced variables is required to understand the extent of influence that hormone therapy has on gray matter volume.

**Table 3.** Reported Hormone Therapy Uses and Benefits

Types of HRT	Reasons that participants engaged in HRT	Reasons that participants did not engage in HRT	Benefits experienced from HRT use
Oral	To minimize menopausal symptoms (*i.e.,* hot flashes, night sweats, vaginal dryness)	No notable symptoms	Minimized menopausal symptoms (*i.e.,* hot flashes, night sweats, vaginal dryness)
Patch	To regulate or minimize menstrual bleeding	Able to cope with symptoms	Regulation and minimization of bleeding
Vaginal	Minimize psychological distress and inconsistent moods	Lack of confidence in treatment	Increased confidence and mood stability
Other (not specified)	To assist with physiological health risks (*i.e.,* blood pressure, low bone density, heart problems)	Not recommended or discussed with physician	Reduced physiological symptoms (*i.e.,* better skin and hair, less aches and pains, fewer headaches and migraines)
	Recommended by physician	Contraindicating health issues	Reduced insomnia
	Used as post-care after hysterectomy		Increased energy
			Reduced risk of osteoporosis and heart disease

The reported types of hormone replacement therapy used by the current sample, the reasons that the participants from the current sample chose to engage in hormone replacement therapy, the reasons that the participants from the current sample chose not to engage in hormone replacement therapy, and the reported benefits that the hormone replacement therapy users experienced.

There are several additional limitations that may have influenced our findings. First, the participants in the current study may not be representative of the population, as they have participated in a, nearly three decades long, cohort study. The individuals who chose to remain and continuously participate in the WHAP might represent a very different sampling than the population at large. Participants who are able to remain might have a vested interest in the aging population, they might be more health conscious, they may have the money and time available to devote to this study instead of working full time, *etc.* Therefore, the participants that comprise the current study might have a significant advantage to health and wellness. In addition to the potential motivations of the study participants that may limit generalizability, the participants of this study were all Caucasian Australian nationals. This further limits the generalizability of the results, even among Australians where there is a diversity that is not represented in the current study. It is critical to include multiple populations in the study of healthy aging including, but not limited to, racial, ethnic, cultural, and socioeconomic diversity, to fully understand the range of healthy aging experiences. A factor that was not accounted for is the time between menopause and the time of imaging; this may have influenced our results as participants would still experience an expected decline of gray matter volume over time. There are also other factors that heavily influence aging and gray matter volume that were not investigated in this study, including activity, genetics, socioeconomic status, and more.^28,29^ These factors could still have impacted our participants and influenced the results that we examined, as risk factors do not exist in a singular vacuum.

There is much work to be done in further examining the impact of endogenous and modifiable estrogen on the aging female brain. In examining the lifetime exposure of estrogen, it will be imperative to accurately assess the influence of endogenous and exogenous influences on lifetime estrogen exposure, including breastfeeding, pregnancies, hormonal contraceptives, surgeries, and hormone therapies. The current study was limited in cohort size and variables to control for; future studies and replication would benefit from more rigorous sampling. Further research should also include randomized control trials and rigorous study designs to demonstrate the impact, if any, of hormone therapy on brain-level correlates. It would also be useful to use a multi-modal approach within the same participants to better understand the impact of estrogen on gray and white matter and cognition. Finally, future research should examine these changes in gray matter volume dependent on modifiable risk factors from a longitudinal perspective. The data shown here represent a snapshot in time but do not account for individual gray matter volume and changes seen on an individual basis over time.

Overall, our results did not demonstrate any significant differences in gray matter volume between HRT users and nonusers, there was a small area in the occipital lobe that demonstrated HRT nonusers group having greater gray matter volume than the HRT, but this did not reach significance. Furthermore, there was no measurable relationship between lifetime estrogen exposure and gray matter volume in HRT nonusers. These findings were generally surprising but may be explained by a combination of participant characteristics and features of study design. The unique circumstances of women’s health and aging remains an understudied portion of the literature and it is critical that we continue to give attention to this portion of our growing aging population.

## Abbreviations Used

EPI: Echo Planar Imaging
FSL: Brain Software Library
HRT: Hormone Replacement Therapy
MPRAGE: Magnetization Prepared Rapid Gradient Echo
MRI: Magnetic Resonance Imaging
TE: Echo Time
TR: Repetition Time
VBM: Voxel-based Morphometry
WHAP: Women's Healthy Ageing Project
